# Identification of CXCL10 as a Prognostic Biomarker for Clear Cell Renal Cell Carcinoma

**DOI:** 10.3389/fonc.2022.857619

**Published:** 2022-02-28

**Authors:** Genyi Qu, Hao Wang, Huiqin Yan, Genlin Liu, Min Wu

**Affiliations:** ^1^ Department of Urology, Zhuzhou Central Hospital, Zhuzhou, China; ^2^ Department of Urology, The First Affiliated Hospital, Hengyang Medical School, University of South China, Hengyang, China; ^3^ Department of Obstetrics, Zhuzhou Central Hospital, Zhuzhou, China; ^4^ Department of Emergency, Zhuzhou Central Hospital, Zhuzhou, China

**Keywords:** clear cell renal cell carcinoma (ccRCC), bioinformatics analysis, differentially expressed genes (DEGs), key gene, survival analysis

## Abstract

**Background:**

One of the widespread forms of kidney tumor is clear cell renal cell carcinoma (ccRCC), with poor prognosis and insensitivity to radio chemotherapy as there is limited capacity to understand the disease mechanism. This study aims at identifying potential biomarkers and the underlying processes of ccRCC using bioinformatics analysis.

**Methods:**

Transcriptome data of relevant samples were downloaded from The Cancer Genome Atlas (TCGA) database. R software was used to screen differentially expressed genes (DEGs) using the “edgeR” package. Two types of analysis—Gene Ontology (GO) functional and Kyoto Encyclopedia of Genes and Genomes (KEGG) pathway enrichment—were accomplished by applying Database for Annotation, Visualization, and Integrated Discovery (DAVID) and Search Tool for the Retrieval of Interacting Genes database (STRING) online bioinformatics tools. A protein–protein interaction (PPI) network of the identified DEGs was constructed using Cytoscape software, and hub genes were subsequently selected *via* the Cytohubba plug-in. The selected genes were input into Oncomine for verification. Finally, selected hub genes were analyzed by doing survival analysis to notice the relationship between survival (OS) rate and the selected genes’ level of expression.

**Results:**

There were 1,855 DEGs found connected to ccRCC, with 1,207 upregulated genes and 648 downregulated genes. G-protein-coupled receptor signaling pathway, integral component of membrane, calcium ion binding, and cytokine–cytokine receptor interaction were among the DEGs discovered. Oncomine confirmed the top six hub genes from the PPI network (C3, CXCR3, CXCL10, CCR5, CCL4, and CCL5). A high level of expression of CXCL10, one of these hub genes, was linked to a poor prognosis in individuals with ccRCC. The results of survival analysis showed that the expression level of CXCL10 was significantly correlated with the prognosis of ccRCC patients (*p* < 0.05).

**Conclusions:**

From the analysis, the following results were drawn: CXCL10 might be a potential prognostic biomarker and novel therapeutic target for ccRCC.

## Introduction

The most common occurring form of kidney tumor is renal cell carcinoma (RCC), which is responsible for almost 3% of malignant tumors in adults. Every year, more than 350,000 new individuals are affected by RCC, and 140,000 deaths occur (1). In addition, a type of RCC is clear cell renal cell carcinoma (ccRCC), which is responsible for almost 75%–80% of cases (2). The typical treatment for ccRCC is surgery, although it has a significant risk of metastasis, a poor prognosis, and is resistant to traditional chemotherapies and radiotherapies (3). With the rapid advancement of molecular biological tools specifically in the last decade, immune checkpoint inhibitors (ICIs) and targeted therapeutics have been explored as promising treatment strategies (4). Though different types of agents have resulted in enhanced patient outcomes, still complete response results are obtained rarely (5). Therefore, exploring the mechanism of ccRCC development and discovering novel biomarkers remain important areas of research. Previous studies have explored the molecular biological mechanism of ccRCC. However, identification of these genes does not fully explain the pathogenesis of ccRCC, making it necessary to explore novel tumor markers so that the progress and development of the disease can be better understood.

Microarray technology is a powerful tool that facilitates concurrent analysis of the expression of thousands of genes and can provide evidence for the mechanism of tumors. Bioinformatics combines computer technology and molecular biology, and can reveal patterns and interactions to provide insight into mechanisms and identify potential genes and paths of interest (6). The development of microarray technology and bioinformatics analysis allow the detection of gene expression levels, facilitating the search for differentially expressed genes (DEGs) (7) and functional pathways associated with tumorigenesis and development of ccRCC (8, 9). In this study, The Cancer Genome Atlas (TCGA) database was used to mine the DEGs of ccRCC. Subsequently, we performed Gene Ontology (GO) and Kyoto Encyclopedia of Genes and Genomes (KEGG) pathway enrichment analysis, followed by protein–protein interaction (PPI) network construction to identify the selected genes. These genes were then verified in the Oncomine database, and we constructed Kaplan–Meier plots for survival analysis. The goal of this research was to explore the specific genes involved in the growth of ccRCC, and the results provide the research basis for targeted precise treatment and prognosis prediction of this common disease.

## Materials and Methods

### Data Collection

The ccRCC sample data were obtained from the database extracted from TCGA (https://cancergenome.nih.gov/) (10) by applying GDC-client. The downloaded genomic data format is FPKM. There were 541 cases of ccRCC and 70 cases of normal renal tissue in the study. For patient characteristics, see **Supplementary Table 1**.

### Identification of DEGs

The “edgeR” package based on the Poisson model in the R language software (version 3.5.3, https://www.r-project.org/) was used to standardize the obtained information and perform differential expression analysis. Genes with adjusted |log fold-change (FC)| > 1.50 values and false discovery rate (FDR) <0.05 were considered DEGs. The “ggplot2” package in R language software was for graphical visualization and data analysis including volcano plot and heat map construction.

### GO Functional and KEGG Pathway Enrichment Analysis

DAVID (https://david.ncifcrf.gov) (11) is an online bioinformatics resource. For the chosen DEGs, the online tool in the DAVID database was selected to be used for performing GO functional and KEGG pathway enrichment analysis. *p*-values of less than 0.05 were deemed statistically significant. For the main functional annotation and visualization of the investigation, the R software package “cluster Profiler” was used.

### PPI Network Construction and Hub Genes Selection

STRING, version 10.5 (https://cn.string-db.org/), was used to identify known and predictable PPIs (11). The PPI network between DEGs was analyzed using STRING database online tools, with a screening criteria of confidence > 0.7. The PPI network was reconstructed by using Cytoscape software (version 3.7.2) (12). The plug-in CytoHubba was used to screen the ten hub genes with the highest connection degree in the PPI network (http://apps.cytoscape.org/apps/CytoHubba).

### Verification of Hub Genes

Oncomine (https://www.oncomine.org) is a sophisticated bioinformatics discovery platform (13). The identified hub genes were examined in the Oncomine database. The search criteria settings were as follows: (1) Gene: hub gene name; (2) Analysis Type: Cancer vs. Normal Analysis; (3) Cancer Type: Clear cell renal cell carcinoma. The cutoff criteria settings were as follows: *p*-value < 1E-4, Fold Change > 2, and Gene Rank=Top 10%. The relevant ccRCC data of the hub genes were obtained in Oncomine, and the meta-analysis was performed with *p* < 0.05 as screening criteria.

### Survival Analysis of Hub Genes

Using R language software, the link between the expression level of hub genes and the overall survival (OS) rate of patients with ccRCC was analyzed by Kaplan–Meier plots. The results were evaluated by log-rank test, and *p* < 0.05 was set as the critical value for screening.

### Statistical Analyses

This study utilized R software (version 4.0.0: http://www.r-project.org) for conducting statistical analyses. Survival analysis was performed using Kaplan–Meier plots, when *p* < 0.05 indicated statistical significance.

## Results

### Identification of DEGs

**Figure 1:**
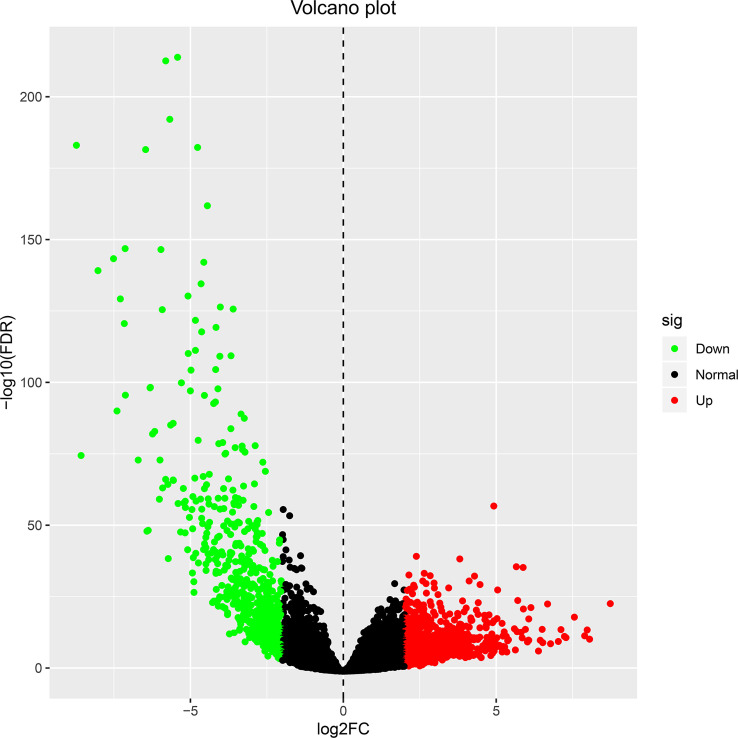
The volcano plot for differentially expressed genes in ccRCC and normal kidney tissue. Genes with adjusted |log FC| > 1.5 and FDR <0.05 values were considered DEGs. Red dots indicate upregulated genes, green dots indicate downregulated genes, and black dots indicate genes without difference. ccRCC, clear cell renal cell carcinoma; FC, fold change; FDR, false discovery rate; DEGs, differentially expressed genes.

**Figure 2:**
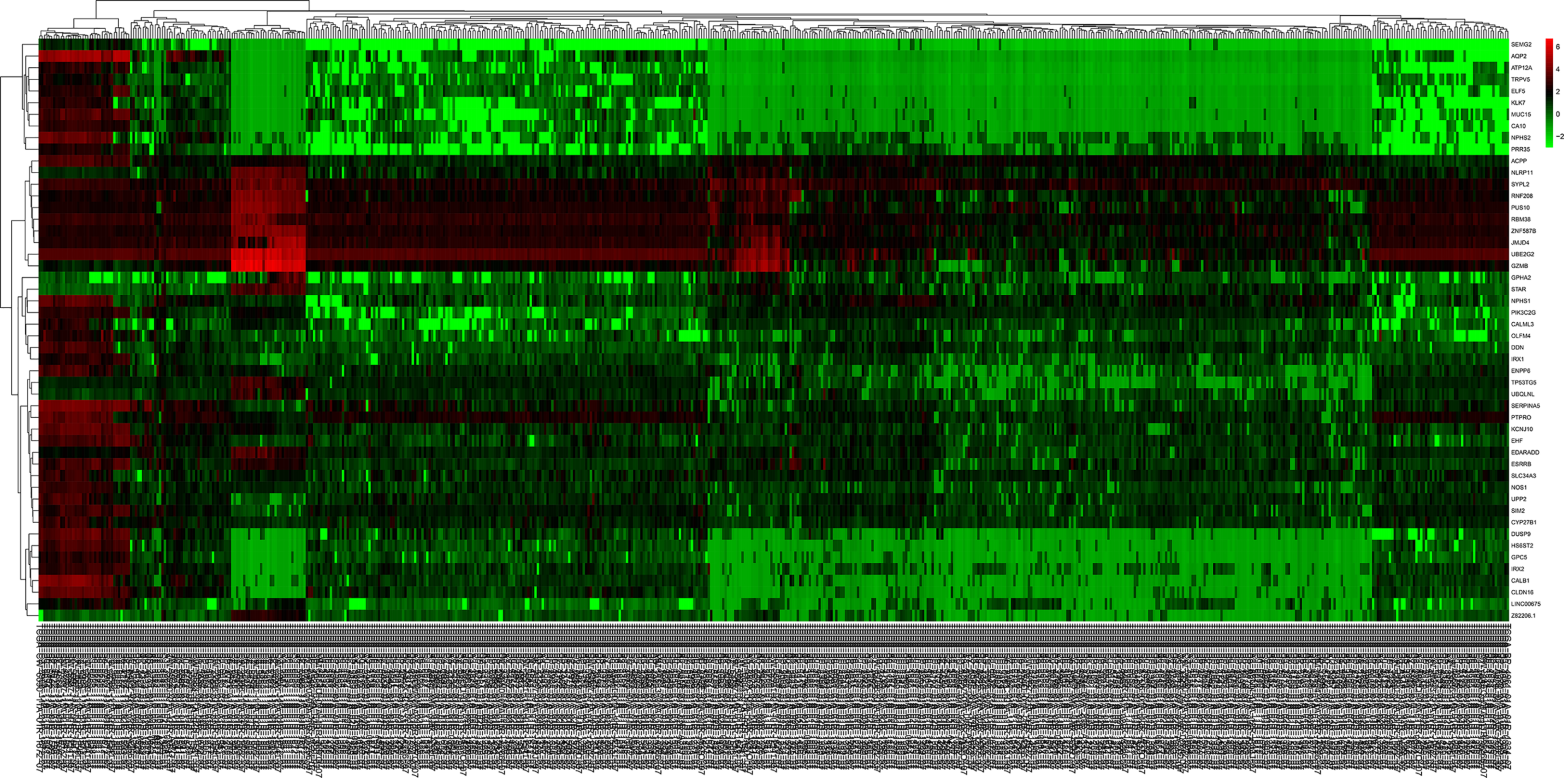
The heat map of the top 50 DEGs in ccRCC and normal kidney tissue. Red squares indicate upregulated genes, green squares indicate downregulated genes, and black squares indicate genes without difference. DEGs, differentially expressed genes; ccRCC, clear cell renal cell carcinoma.

Transcriptome data of ccRCC were taken from TCGA directory and included 541 samples of ccRCC and 70 samples of normal kidney tissue. We normalized and logarithmized the data, removed all probes without corresponding gene annotation information, and removed all repeated probes using R language software. Finally, the expression profiles of 18,764 genes and 611 samples were extracted. Using the “edgeR” package, 1,855 DEGs were selected, which included 1,207 (**Supplementary Table 2**) and 648 (**Supplementary Table 3**) upregulated and downregulated genes, respectively. To visualize the results, a volcano plot and heat map were designed with the help of “ggplot2” package in R of the top 50 DEGs with the most significant differences, as displayed in **Figures 1** and **2**, respectively.

### GO Functional and KEGG Pathway Enrichment Analysis

**Figure 3:**
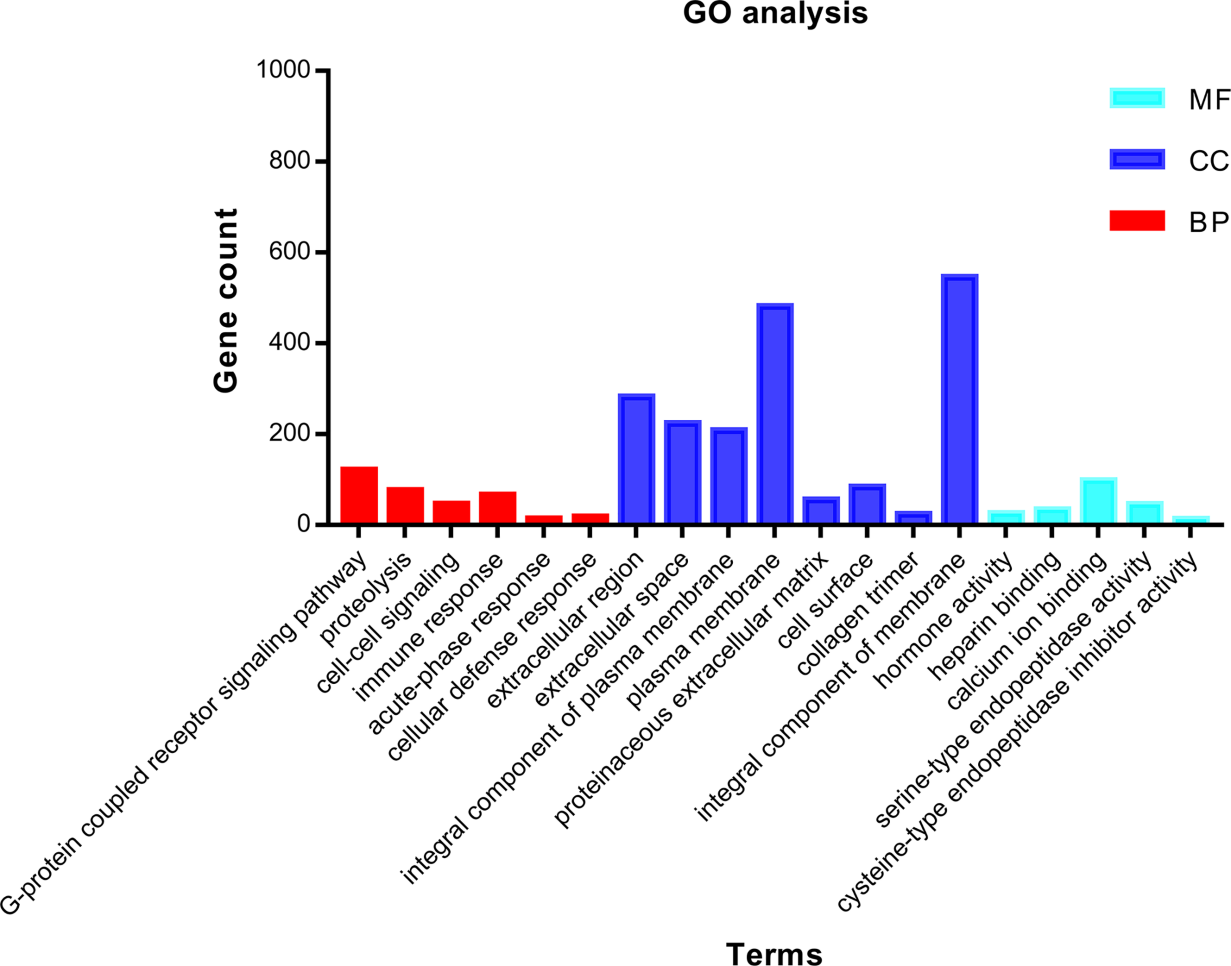
GO functional enrichment analysis of 1,855 DEGs associated with ccRCC. Bright blue columns represent MF, navy blue columns represent CC, and red columns represent MF. GO, Gene Ontology; CC, cellular component; MF, molecular function; BP, biological process; DEGs, differentially expressed genes; ccRCC, clear cell renal cell carcinoma.

In order to grasp the biological functions of the 1,855 identified DEGs, the data of genes were entered in the online tool in DAVID to perform GO functional and KEGG pathway enrichment analysis. GO functional enrichment analysis includes biological process (BP), cell composition (CC), and molecular function (MF) (14). For BP, the DEGs were largely enriched in G-protein-coupled receptor signaling pathway, proteolysis, immune response, cell–cell signaling, acute-phase response, and cellular defense response. For CC, the DEGs were largely enriched in extracellular region, integral component of plasma membrane, collagen trimer, plasma membrane, proteinaceous extracellular matrix, cell surface, and integral component of membrane. For MF, the DEGs were largely enriched in hormone activity, heparin binding, calcium ion binding, serine-type endopeptidase activity, and cysteine-type endopeptidase inhibitor activity. The main terms of GO functional analysis are displayed in **Table 1** and were further visualized by applying the “clusterProfiler” package in R software, as displayed in **Figure 3**.

**Table 1:** GO functional enrichment analysis of 1,855 DEGs associated with ccRCC.

Category	Term	Description	Count	*p*-value
BP	GO:0007186	G-protein-coupled receptor signaling pathway	121	4.33E-06
BP	GO:0006508	Proteolysis	76	5.45E-06
BP	GO:0007267	Cell–cell signaling	46	6.56E-06
BP	GO:0006955	Immune response	66	8.84E-06
BP	GO:0006953	Acute-phase response	14	1.95E-05
BP	GO:0006968	Cellular defense response	18	2.04E-05
CC	GO:0005576	Extracellular region	282	1.21E-29
CC	GO:0005615	Extracellular space	224	1.90E-20
CC	GO:0005887	Integral component of plasma membrane	208	6.06E-13
CC	GO:0005886	Plasma membrane	481	4.92E-11
CC	GO:0005578	Proteinaceous extracellular matrix	55	1.41E-08
CC	GO:0009986	Cell surface	84	1.14E-06
CC	GO:0005581	Collagen trimer	24	5.05E-06
CC	GO:0016021	Integral component of membrane	545	5.77E-06
MF	GO:0005179	Hormone activity	25	1.14E-06
MF	GO:0008201	Heparin binding	34	2.45E-06
MF	GO:0005509	Calcium ion binding	98	7.92E-06
MF	GO:0004252	Serine-type endopeptidase activity	45	8.86E-06
MF	GO:0004869	Cysteine-type endopeptidase inhibitor activity	13	1.65E-05

GO, Gene Ontology; DEGs, differentially expressed genes; ccRCC, clear cell renal cell carcinoma.

**Figure 4:**
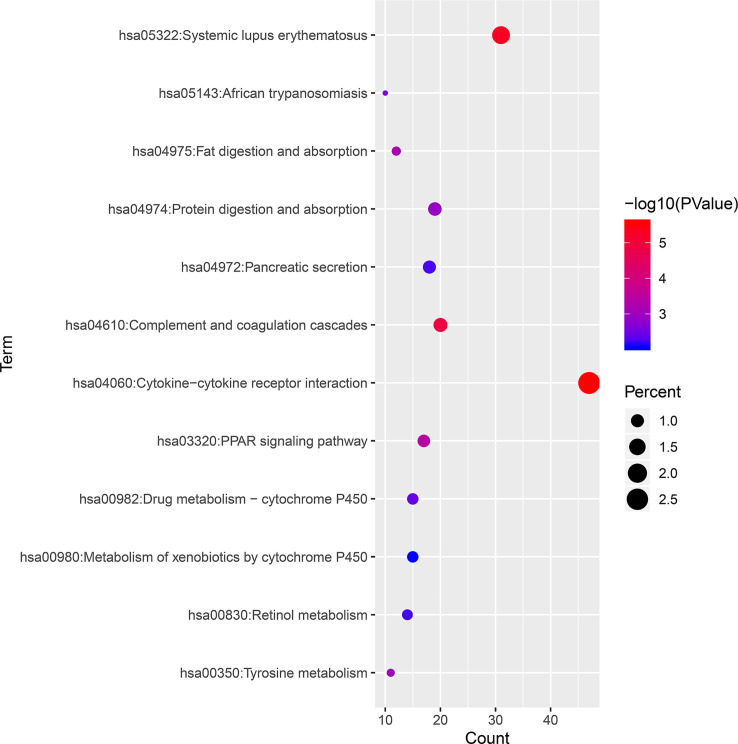
KEGG pathway enrichment analysis of 1,855 DEGs associated with ccRCC. KEGG, Kyoto Encyclopedia of Genes and Genomes; ccRCC, clear cell renal cell carcinoma; DEGs, differentially expressed genes.

KEGG pathways were largely enriched in cytokine–cytokine receptor interaction, systemic lupus erythematosus, complement and coagulation cascades, PPAR signaling pathway, fat digestion and absorption, tyrosine metabolism, protein digestion and absorption, African trypanosomiasis, drug metabolism–cytochrome P450, pancreatic secretion, retinol metabolism, and metabolism of xenobiotics by cytochrome P450. The main terms of KEGG pathway enrichment analysis are displayed in **Table 2** and were further visualized by applying the “clusterProfiler” package in R software, as displayed in **Figure 4**.

**Table 2:** KEGG pathway enrichment analysis of 1,855 DEGs associated with ccRCC.

Category	Term	Description	Count	*p*-value
KEGG	hsa04060	Cytokine–cytokine receptor interaction	47	2.73E-06
KEGG	hsa05322	Systemic lupus erythematosus	31	5.16E-06
KEGG	hsa04610	Complement and coagulation cascades	20	1.30E-05
KEGG	hsa03320	PPAR signaling pathway	17	3.82E-04
KEGG	hsa04975	Fat digestion and absorption	12	7.00E-04
KEGG	hsa00350	Tyrosine metabolism	11	0.001092
KEGG	hsa04974	Protein digestion and absorption	19	0.001233
KEGG	hsa05143	African trypanosomiasis	10	0.002745
KEGG	hsa00982	Drug metabolism - cytochrome P450	15	0.003911
KEGG	hsa04972	Pancreatic secretion	18	0.005701
KEGG	hsa00830	Retinol metabolism	14	0.006019
KEGG	hsa00980	Metabolism of xenobiotics by cytochrome P450	15	0.008536

KEGG, Kyoto Encyclopedia of Genes and Genomes; DEGs, differentially expressed genes; ccRCC, clear cell renal cell carcinoma.

### PPI Network and Results of Selected Hub Genes

**Figure 5:**
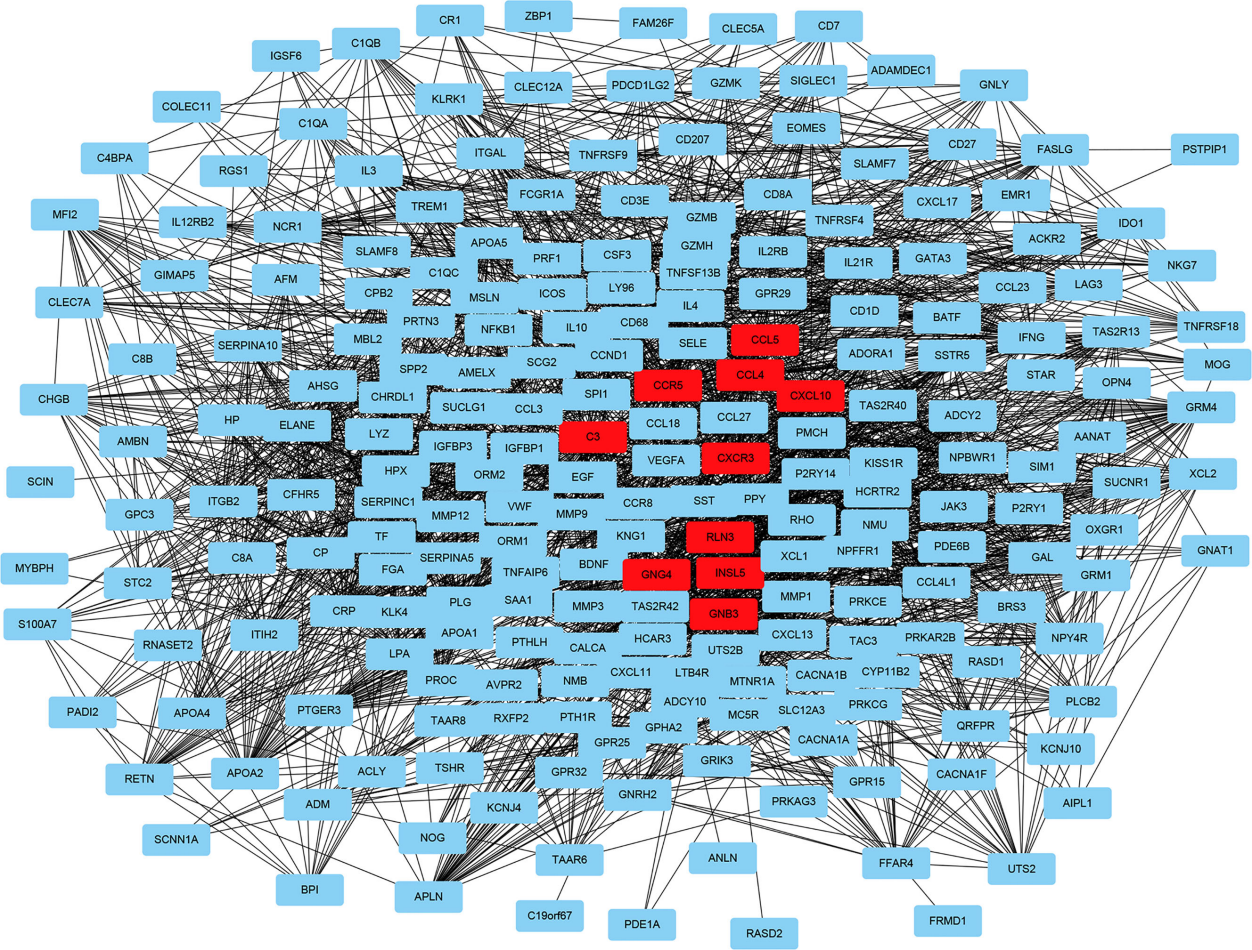
Reconstructed protein–protein interaction network using Cytoscape. The ten hub genes with the highest connection degree are indicated in red.

**Figure 6:**
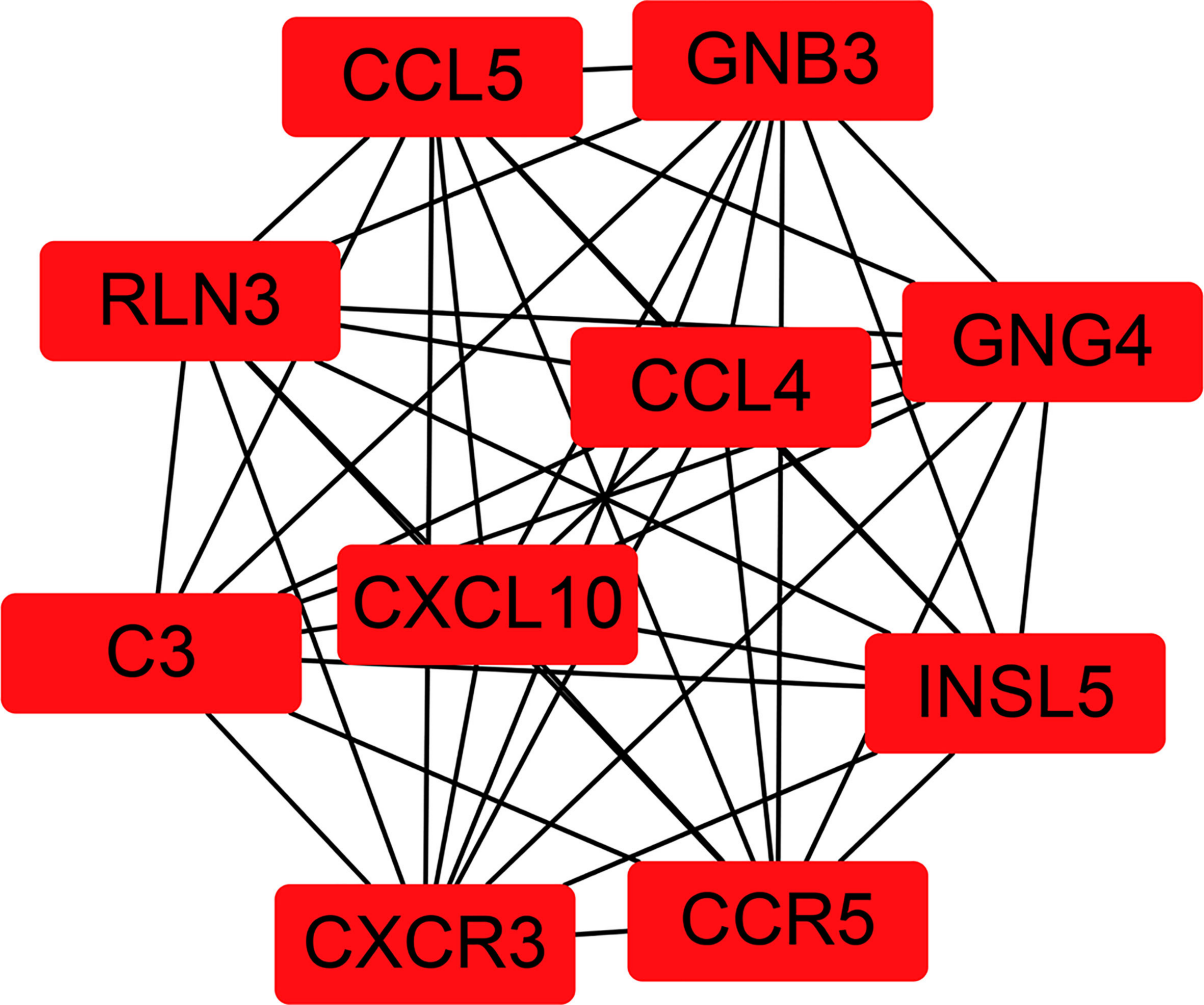
The 10 most connected hub genes selected from the PPI network.

To fully understand the connection of the 1,855 DEGs, STRING’s web tool was used to enter the discovered genes. PPI network was reconstructed by Cytoscape software using STRING (**Figure 5**). The ten selected genes were chosen who had the highest connectivity with the plug-in cytoHubba, and included complement C3 (C3), C-X-C chemokine receptor type 3 (CXCR3), C-X-C motif chemokine ligand 10 (CXCL10), C-C chemokine receptor type 5 (CCR5), C-C motif Chemokine Ligand 4 (CCL4), Chemokine Ligand 5 (CCL5), Insulin-like peptide 5 (INSL5), G-protein subunit gamma 4 (GNG4), G-protein subunit beta 3 (GNB3), and relaxin-3 (RLN3), as shown in **Figure 6**.

### Verification of hub genes in Oncomine

**Figure 7:**
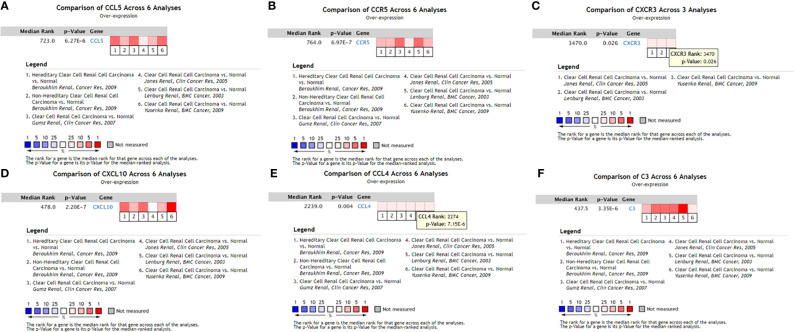
Expression levels of six hub genes of the PPI network verified in Oncomine. **(A)** CCL5 meta-analysis results in the Oncomine database; **(B)** CCR5 meta-analysis results in the Oncomine database; **(C)** CXCR3 meta-analysis results in the Oncomine database; **(D)** CXCL10 meta-analysis results in the Oncomine database; **(E)** CCL4 meta-analysis results in the Oncomine database; **(F)** C3 meta-analysis results in the Oncomine database. ccRCC, clear cell renal cell carcinoma.

The data obtained from Oncomine included the gene expression of identified hub genes. Six of the ten hub genes were verified in Oncomine, namely, C3, CXCR3, CXCL10, CCR5, CCL4, and CCL5. **Figure 7** shows the results obtained from the meta-analysis.

### Survival Analysis

**Figure 8:**
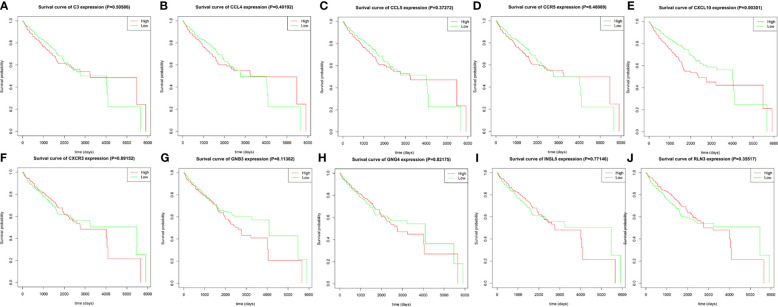
The prognostic value of the ten identified hub genes for overall survival of patients with ccRCC. **(A)** Kaplan–Meier plot for C3, *p* = 0.50586; **(B)** Kaplan–Meier plot for CCL4, *p* = 0.40192; **(C)** Kaplan–Meier plot for CCL5, *p* = 0.37272; **(D)** Kaplan–Meier plot for CCR5, *p* = 0.48089; **(E)** Kaplan–Meier plot for CXCL10, *p* = 0.00301; **(F)** CXCR3, *p* = 0.89152; **(G)** Kaplan–Meier plot for GNB3, *p* = 0.11382 **(H)** Kaplan–Meier plot for GNG4, *p* = 0.82175; **(I)** Kaplan–Meier plot for INSL5, *p* = 0.77146; **(J)** Kaplan–Meier plot for RLN3, *p* = 0.35517. A value of *p* < 0.05 was considered statistically significant.

Using R language software for critical analysis of link between hub genes expression and the associated OS rate of ccRCC, Kaplan–Meier plots were used. For the ten hub genes, only increased expression level of CXCL10 was associated with a worse survival rate for ccRCC patients. All results are displayed in **Figure 8**.

## Discussion

As one of the most common malignant tumors, ccRCC is also the most common kidney tumor accounting for roughly 3% of adult cancers (1, 15). Typical symptoms of ccRCC include hematuria, low back pain, and a mass in the upper abdomen or waist, but only few patients present these symptoms, limiting detection of ccRCC before reaching an advanced stage. This cancer is characterized by high incidence, poor prognosis, and insensitivity to radiotherapy and chemotherapy (3). The tumorigenesis and development of ccRCC are complicated processes, with many factors contributing to the development and metastasis of ccRCC, so the details of the specific mechanism remain unclear. Although some significant biomarkers were identified in previous studies, including VHL, PBRM1, and BAP1 (16), the mutation of von Hippel–Lindau (VHL), which is a tumor suppressor gene, has played a significant role in the tumorigenesis of ccRCC (16, 17). VHL is involved in the degradation of hypoxia-inducible factor (HIF) protein, and VHL inactivation leads to abnormal aggregation of HIF protein, leading to an imbalance of HIF-targeted genes that regulate angiogenesis, glycolysis, and apoptosis (16). Recent studies have also found that (Polybromo 1) PBRM1 and (BRCA1-associated protein 1) BAP1 mutations can act in the development of ccRCC (18–20). These are not sufficient to understand the development and prognosis of ccRCC. Therefore, there is a significant need to discover more novel biomarkers, which may facilitate earlier diagnosis, more effective treatment, and improved prognosis of ccRCC. TCGA is a project that was jointly initiated by the American Cancer Center and the American Human Genome Research Center in 2006. TCGA utilizes high-throughput sequencing data to build a comprehensive, multi-dimensional cancer map, to elucidate occurrence, diagnosis, treatment, and pathogenesis of cancer (21). Traditional molecular biology experiments can only study the function of a few genes at the same time. Thus, mining the TCGA database can find patterns in a large amount of biological information.

In our study, bioinformatics technology was used to mine ccRCC genome data downloaded from TCGA. In total, 1,855 DEGs were found in ccRCC, including 1,207 upregulated genes and 648 downregulated genes. GO functional and KEGG pathway enrichment analysis were performed to better understand the relevant biological functions of the DEGs. GO functional enrichment analysis revealed that the 1,855 DEGs were mainly enriched in 19 terms, namely, G-protein-coupled receptor signaling pathway, proteolysis, immune response, cell–cell signaling, acute-phase response, cellular defense response, proteinaceous extracellular matrix, extracellular space, integral component of plasma membrane, calcium ion binding, extracellular region, cell surface, collagen trimer, plasma membrane, integral component of membrane, hormone activity, heparin binding, serine-type endopeptidase activity, and cysteine-type endopeptidase inhibitor activity. KEGG pathway enrichment analysis revealed that the 1,855 DEGs were mainly enriched in 12 pathways, namely, fat digestion and absorption, cytokine–cytokine receptor interaction, complement and coagulation cascades, systemic lupus erythematosus, African trypanosomiasis, PPAR signaling pathway, tyrosine metabolism, protein digestion and absorption, drug metabolism–cytochrome P450, pancreatic secretion, retinol metabolism, and metabolism of xenobiotics by cytochrome P450. Subsequently, the online tool in STRING and Cytoscape were used to construct a PPI network of the identified DEGs, and the ten hub genes with the highest connection degree in the PPI network were selected, namely, C3, CXCR3, CXCL10, CCR5, CCL4, CCL5, INSL5, GNG4, GNB3, and RLN3. To verify the results, these ten hub genes were input to the Oncomine database. Six of these hub genes (C3, CXCR3, CXCL10, CCR5, CCL4, and CCL5) were subjected to meta-analysis, but the data about the other four hub genes (INSL5, GNG4, GNB3, and RLN3) were not found in Oncomine. Additional study will be required to confirm the role of these four genes in ccRCC.

Complement C3 is an important component of complement activation. C3 activation results in the production of C3a. C3aR, as the receptor of C3a, is expressed in many immune cells (22). Tumor cell-derived C3a is an important cancer-promoting factor in TME and can enhance immunosuppression to promote tumor cell growth *via* the C3a-C3aR signaling pathway, providing a potential target for immune checkpoint block (ICB) therapy (23). In the development of breast cancer (24) and lung cancer (23), the C3a-C3aR signaling pathway plays a key role. CXCR3 has seven transmembrane domains. It is expressed on Th1, CTL, NK, and NKT cells, and is regulated by IFN-γ (25). Cancer cells can also express CXCR3, and Monteagudo et al. (24) described that the level at which CXCR3 is expressed in malignant melanoma was related to metastatic potential and the prognosis of patients. CCL5, also known as RANTES, is a member of the C-C chemokine family that is expressed in a wide range of immune cells. Its role in tumor-related formation mechanisms is unclear, because it aids in the elicitation of anti-tumor immune responses, but also can stimulate tumor progression and metastasis (26). CCR5 is a cell membrane protein of the G protein-coupled factor superfamily (GPCR). As a receptor encoding CCL5, it can promote tumorigenesis, matrix formation, and tumor development. CCL5/CCR5 activates nuclear factor-κB through the PI3K/AKT signaling pathway, the effects of mitogen-activated protein kinase and extracellular regulated protein kinases (ERK), leading to activation of αvβ3 integrin and promoting cell migration (27–29). CCL5/CCR5 is a biomarker for poor prognosis of pancreatic cancer (31), prostate cancer (31), lung cancer (29), and ovarian cancer (28). CCL4 belongs to the proinflammatory C-C subfamily and acts in inflammation, immune regulation, and tumor progression. CCL4 is involved in the proliferation and metastasis of various cancers such as breast cancer (31) and squamous cell carcinoma (31). In addition, Fang et al. (32) found that epithelial-to-mesenchymal transition (EMT) induced by CCL4 in RWPE-1 cells can ultimately lead to the development of tumor in prostate. There has been no previous investigation of the roles of C3, CXCR3, CCL4, CCL5, or CCR5 in the development of ccRCC, and these genes should be investigated in future research.

To further analyze the relationship between the level of expression of the ten most-connected hub genes and the corresponding OS rate of ccRCC, survival analysis was performed. Only increased gene expression of CXCL10 was significantly associated with a worse survival rate for ccRCC patients. C-X-C motif chemokine ligand 10 (CXCL10) is also called interferon gamma-inducible protein 10 (IP-10), and is a 10-kDa secreted protein in the CXC subfamily of cytokines. This protein participates in leukocyte trafficking and regulates adaptive immune, inflammatory, hematopoietic, and angiogenic processes (33). CXCL10 is a selective ligand for CXCR3, and the CXCL10/CXCR3 signaling pathway regulates leukocyte trafficking and angiogenesis through paracrine interactions, while signaling of autocrine CXCL10/CXCR3 in tumor cells can promote growth of cancer and metastasis by regulating cell adhesion, invasion, and migration capabilities (34, 35). There are also studies associating the CXCL10/CXCR3 gene expression pathway with enhanced metastatic potential and poor prognosis in patients with melanoma (36) and colon cancer (37). Polimeno et al. (38) discovered that when the results were contrasted with the control group, which was healthy, there was a higher level of CXCL10 in the serum of RCC patients. Another study from Japan found that CXCR3 was associated with metastasis of RCC (39). Thus, CXCR3 could be a new therapeutic target and predictive biomarker for ccRCC.

The goal of this research was to apply bioinformatics analysis for the identification of a key DNA segment that is involved in the ccRCC development. The results of TCGA showed 6 hub genes and 1,855 DEGs. Of the identified hub genes, only CXCL10 expression was linked to a worse outcome in ccRCC patients. However, the function of this gene remains to be characterized experimentally, and further in-depth study is needed to clarify the specific biological functions of CXCL10 in the mechanism of ccRCC. The results presented here should provide new clues and directions for the early diagnosis, prognosis prediction, and treatment of ccRCC.

## Data Availability Statement

The datasets presented in this study can be found in online repositories. The names of the repository/repositories and accession number(s) can be found in the article/**Supplementary Material**.

## Author Contributions

GQ and HW wrote the main manuscript text. GL performed experiments. HY and MW collected data. All authors contributed to the article and approved the submitted version.

## Funding

The Hunan Natural Science Foundation (#S2021JJQNJJ0018) provided funding for this research.

## Conflict of Interest

The authors declare that the research was conducted in the absence of any commercial or financial relationships that could be construed as a potential conflict of interest.

## Publisher’s Note

All claims expressed in this article are solely those of the authors and do not necessarily represent those of their affiliated organizations, or those of the publisher, the editors and the reviewers. Any product that may be evaluated in this article, or claim that may be made by its manufacturer, is not guaranteed or endorsed by the publisher.
